# Experimental Performance Analysis of a Pilot-Scale Biomass-Assisted Recirculating Mixed-Flow Dryer for Drying Paddy

**DOI:** 10.1155/2022/4373292

**Published:** 2022-01-27

**Authors:** M. Yahya, Hendriwan Fahmi, R. Hasibuan

**Affiliations:** ^1^Fakultas Teknologi Industri, Institut Teknologi Padang, Indonesia; ^2^Departemen Teknik Kimia, Universitas Sumatera Utara, Indonesia

## Abstract

A large amount of heat energy is required for paddy drying processes to evaporate water from paddy grains. Currently, fossil fuels are being used as an energy source to heat air during the drying process. However, fossil fuels cause air pollution, climate change, and disruption of ecological balance. In this study, to reduce the dependence on fossil fuels for paddy drying, a pilot-scale biomass-assisted recirculating mixed-flow drying system (PSBA-RMFD) for drying paddy was designed, installed, and tested. In this PSBA-RMFD, the heat energy required for heating the drying air was provided only by biomass. The PSBA-RMFD comprises a biomass furnace, drying column, vibratory feeder, bucket elevator, and blower. This study is aimed at evaluating the performance of the PSBA-RMFD with a drying capacity of 400 kg/h. The performance metrics of the PSBA-RMFD were specific energy consumption (SEC), specific thermal energy consumption (STEC), specific moisture evaporation rate (SMER), thermal efficiency of the PSBA-RMFD, exergy efficiency of the drying section, and improvement potential of the dryer. From the experiments conducted in this study, the values of the aforementioned performance parameters were as follows: 0.806-8.656 kW h/kg of water evaporated; 0.385-4.136 kW h/kg of water evaporated; 0.122-1.308 kg of water evaporated/kW h; 7.82-83.99%; 15.28-25.64%; and 858.90-1355.62 W, respectively. The paddy moisture content was reduced from 20.90% wet basis (initial weight of 400 kg) to 13.30% wet basis (final weight of 364 kg) in 270 min, with an average temperature of 78.15°C and average relative humidity of 8.55%. The percentage of biomass energy used in the drying system was approximately 47.77% of the overall energy. In addition, the payback period of the PSBA-RMFD was 1.9 years.

## 1. Introduction

Indonesia is an agricultural economy-based country with a vast agricultural land and is one of the world's leading paddy producers. Its paddy production in 2019 reached approximately 84 million t. Indonesia is also one of the world's largest paddy consumers, with an annual per capita consumption of approximately 111.58 kg [1]. In Indonesia, the paddy after being harvested must be dried immediately to avoid mold growth and yellowing of grains, as Indonesia's climate is characterized by high humidity. The harvested paddy contains a high degree of moisture, ranging from 20% to 27% (wet basis). Therefore, to secure a long shelf life, the paddy is typically dried to a moisture content of 14% (wet basis) [2]. Traditionally, paddy is dried using open sun drying, where the product is exposed directly to the sun. Drying with open sunlight has several advantages, including free and abundant supply of energy from the sun, simplicity of the method, and low initial and maintenance costs; however, its disadvantages include, the requirement of a large area, which is a function of the solar radiation, long drying times, lowered quality of the dried products, and significant loss of paddy during the drying process.

To overcome the limitations of open sun drying for drying paddy, a mixed-flow dryer can be used, which offers significant advantages, such as suitability for drying of grains, short drying time, good quality of the dried products, and smaller losses in the quantity of paddy during the drying process [3].

Mixed-flow dryers have been used to dry high-moisture grain products, such as rice [4], maize [5], wheat [6–11], soybeans [12, 13], barley, oats [14], and corn [15]. However, fossil fuels are still being used as energy sources for heating the drying air during the drying process. Fossil fuels suffer from disadvantages, such as generation of significant amounts of CO_2_, SO_2_, and Nox emissions. They cause air pollution, climate change, and disruption of ecological balance. Moreover, their prices are high and will continue to rise, while their resources are limited [16, 17].

Biomass energy serves as an alternative to fossil fuels and has some advantages, such as low emissions and sustainable renewable energy sources. Moreover, it is locally available in abundance and is relatively low-priced.

A few researchers have developed various drying systems using biomass energy sources to dry diverse products, for example, integrating the drying system with a biomass furnace (biomass burner); using fluidized bed dryers to dry paddy [17, 18], convective tray dryers to dry plantain slices [19], maize [20], candle nuts [21], red chili [22], natural rubber sheet [23], cashew kernel [24], food and fish [25], chili [26], pineapple [27], ginger, turmeric guduchi [28], and pineapple; and using tunnel dryers to dry fish [29] and green pepper [30].

Paddy drying process consumes a large amount of heat energy to evaporate water in the paddy grains [31]. Several researchers have conducted performance analyses in terms of energy consumption (specific energy) for different types of dryers for paddy drying; these include among others, fixed bed dryers [32], fixed deep bed dryers [33], inclined bed dryers [34], fluidized bed dryers [17, 18, 35–43], spouted bed dryers [40, 44], crossflow dryers [45, 46], mixed-flow dryers [47], recirculating grain dryers [48], rotary dryers [32, 49], impinging stream dryers [50], and combined impinging stream and pneumatic dryers [51]. The energy consumption of various dryers used for paddy drying is summarized in Table 1.

However, to the best of our knowledge, the performance of a pilot-scale biomass-assisted recirculating mixed-flow dryer (PSBA-RMFD) for drying paddy using biomass energy as a source of heat energy has not yet been reported. In addition, Indonesia produces significantly large amounts of biomass energy every year (approximately 236 million t; 756.083 million GJ), and this can be used as a source of heat energy in the drying process [52]. Therefore, this study is aimed at designing, installing, and evaluating the performance of a PSBA-RMFD for drying paddy using biomass as a source of heat energy.

## 2. Materials and Methods

### 2.1. Experimental Set-Up

**Figure 1:**
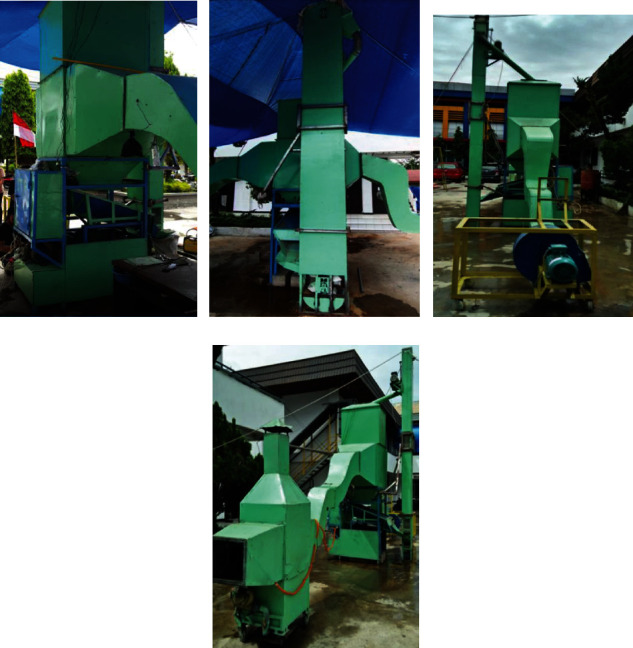
Photograph of the PSBA-RMFD: (a) front view, (b) back view, (c) left view, and (d) right view.

**Figure 2:**
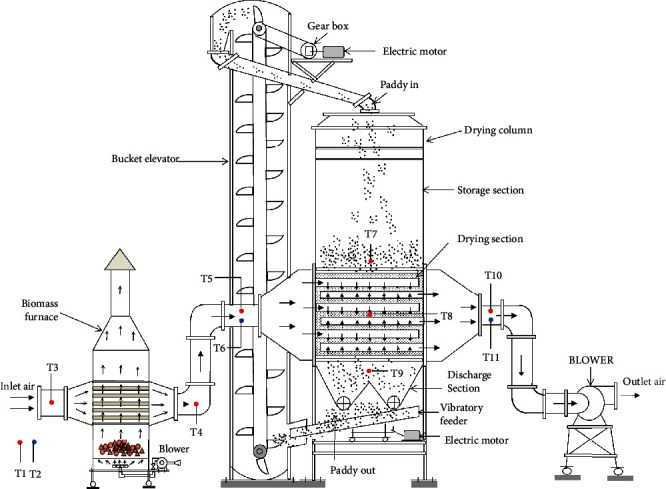
Schematic of the PSBA-RMFD.

**Figure 3:**
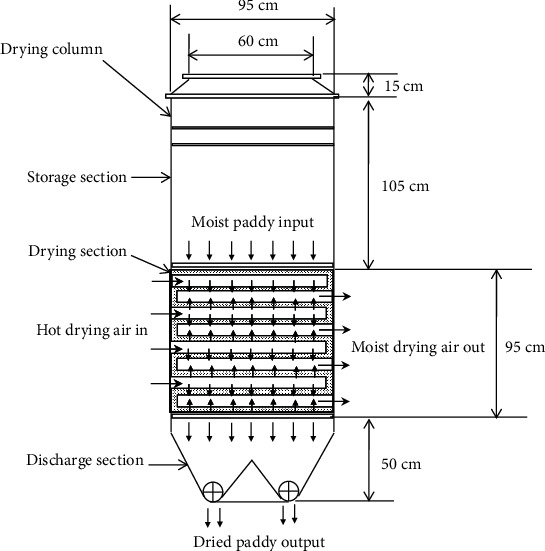
Schematic of the drying column with dimensions.

**Figure 4:**
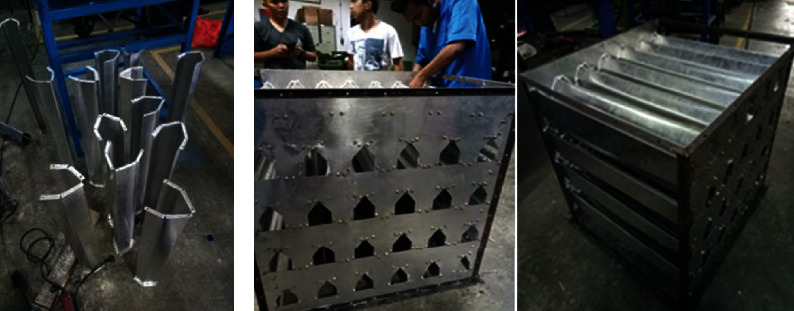
(a) Photograph of the air duct. (b) Photograph of the drying section.

**Figure 5:**
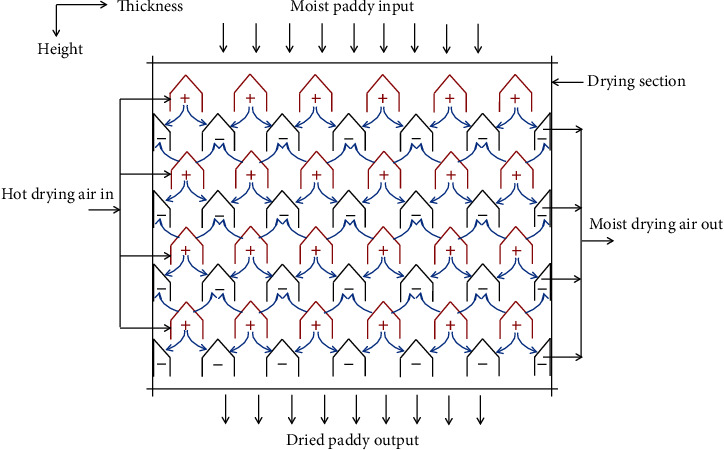
Air duct arrangement in the drying section (+ hot air inlet; – moist air outlet).

**Figure 6:**
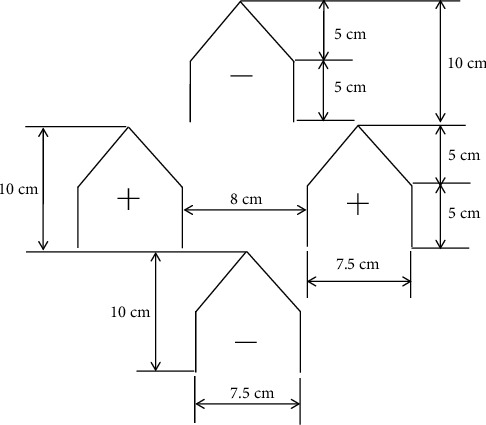
Schematic of the air ducts with dimensions.

A PSBA-RMFD, which comprised a biomass furnace, drying column, vibratory feeder, bucket elevator, and blower, was designed and installed. Photographs and a schematic of the PSBA-RMFD are presented in Figures 1 and 2. The biomass furnace consisted of the following key parts: a furnace chamber, heat exchanger pipes, chimney, and blower. The wall of the furnace chamber was fabricated from Sk-34 fire brick, Sk-34 cement mortar, and AISI 1030 steel plate materials. Sk-34 fire brick has advantages such as high mechanical strenght, high temperature resistance, and high resistance to abrasion; Sk-34 cement mortar has advantages such as high temperature resistance and high adhesiveness. Meanwhile, AISI 1030 steel plate has advantages such as high mechanical strenght and high temperature resistance. The pipes of the heat exchanger were fabricated from mild steel. The drying column had three main sections, namely, the storage, drying, and discharge sections, and its dimensions are shown in Figure 3. The discharge section consisted of a motor, gear box, sprocket, chain, roller, and support structure. The vibratory feeder consisted of a motor, pulley, bearing, trough, and support structure. The bucket elevator consisted of a pulley, belt, buckets, gear box, motor, feed hopper, discharge sprout, and casing. The drying section of the drying column had a cross-sectional area of 95 × 95 cm^2^ and was equipped with inlet and outlet air ducts arranged horizontally. The inlet and outlet air ducts consisted of four rows each. Each inlet air duct row had six full-air ducts, and each outlet air duct row had five full and two half-air ducts. The cross-sectional area of each air duct was 56.25 cm^2^. The inlet and outlet air ducts were wedge-shaped. The photographs of air duct and drying section are shown in Figure 4. Meanwhile, the arrangement of air ducts and their dimensions (in the drying section) are shown in Figures 5 and 6.

The working procedure of the PSBA-RMFD is presented in Figure 2. At first, the dryer is filled with moist paddy by a bucket elevator into the hopper at top the dryer and flowed vertically downwards by gravity, while the discharge roller at the bottom of the dryer is turned off (discharge device is closed). The discharge roller operated on the principle of a rotary valve to allow a constant product mass flow rate. When the moist paddy in the dryer reach the desired height, then the ambient air is passed through the biomass furnace heat exchanger by a centrifugal blower to increase its temperature. The ambient air is heated up to a prescribed temperature with the heat generated by burning the biomass fuel (coconut shell charcoal) in the combustion chamber of the biomass furnace. Then, the heated air is passed to the drying section, from where the heated air is forced through the paddy grains outwards carrying the moisture from drying material. Then, the moist air is discharged into the environment. In the drying section, the moist grains crosses the drying section nearly vertically while heated air (drying air) flows horizontally. Then, the discharge roller is turned on (discharge device is opened). Subsequently, the dried paddy falls into the vibratory feeder, then flow to the bottom of the bucket elevator. From here, the dried paddy is moved or circulated to the top of dryer by the bucket elevator for further drying process. This process continues until the desired moisture content of the paddy grains is achieved.

### 2.2. Experimental Procedure

A drying experiment was conducted to evaluate the performance of the PSBA-RMFD, with a holding capacity of 400 kg. Fresh paddy was purchased from a farmer, and during the drying experiment the ambient temperature, the air temperatures at the entry and exit of the biomass furnace, drying section, and the temperatures of the paddy at various points inside the drying section were measured using thermocouples (type T, accuracy: ±0.1°C, operating range: –200 to 400°C, Japan). An anemometer (model HT383, accuracy: ±0.2 ms^−1^, operating range: 0–30 ms^−1^, China) was used to measure the air velocity at the inlet and outlet of the drying section. The ambient temperature, air temperature, and temperature of the paddy were recorded using a data logger (model AH4000, accuracy: ±0.1°C, channel 12, Japan). A grain moisture tester (type digital, model OEM MC-7828G, accuracy: ±0.5%, measuring range: ±0%–50%, China) was used to measure the change in the moisture content of the paddy. The mass of biomass fuel (coconut shell charcoal) was weighed using a weighing scale (model TKB-0.15, accuracy: ±0.05 kg, measuring range: 0–15 kg, China). The mass of the paddy was weighed using a weighing scale (model Camry, accuracy: ±0.1 kg, measuring range: 0–100 kg, China). The ambient temperature, air temperatures, temperature of the paddy, and moisture content of the paddy were recorded every 30 min. The uncertainty of measurements was determined using the following equation [53, 54]:

(1) $$\begin{array}{c} W_{r}={\left[{\left(\frac{\partial R}{\partial x_{1}}w_{1}\right)}^{2}+{\left(\frac{\partial R}{\partial x_{2}}w_{2}\right)}^{2}+\cdots +{\left(\frac{\partial R}{\partial x_{n}}w_{n}\right)}^{2}\right]}^{1/2}, \end{array}$$

where *W*_*R*_ is the total uncertainty in the result measurement; *x*_1_, *x*_2_, ⋯*x*_*n*_ are the independent variables; and *w*_1_, *w*_2_, ⋯*w*_*n*_ are the corresponding uncertainties in the aforementioned independent variables.

### 2.3. Performance Analysis

The performance of the PSBA-RMFD, in terms of the drying rate $\;\left({\dot{m}}_{\text{water}}\right)$, specific energy consumption (SEC), specific thermal energy consumption (STEC), specific electrical energy consumption (SEEC), specific moisture evaporation rate (SMER), thermal efficiency of the dryer (*η*_th_), exergy efficiency of the drying sections (*η*_Ex_), improvement potential (IP), and efficiency of the biomass furnace (*η*_BF_)  was determined using the equations presented in Table 2. The data obtained from the performance analysis of the PSBA-RMFD during the drying experiment of the paddy are presented in Table 3.

### 2.4. Economic Analysis

For evaluating the economy of a drying system, two important aspects should be considered: first, the investment cost (*I*_*C*_); second, the production cost (*C*_pr_), profit (PR), return on capital (ROC), payback period (PP), and net present value (NPV). *I*_*C*_ of the PSBA-RMFD includes the costs of the biomass furnace, drying column, vibratory feeder, bucket elevator, air duct, motor, blower, and labor for construction and installation. The components of *I*_*C*_ are listed in Table 4. *C*_pr_, PR, ROC, PP, and NPV of the PSBA-RMFD were calculated using the equations presented in Table 5.

## 3. Results and Discussion

### 3.1. Performance Evaluation

Table 6 summarizes the uncertainties in the parameters during the paddy drying experiment. Referring to Table 3, the ambient temperature (T1) and ambient relative humidity ranged between 30.30 and 35.60°C and 50.76% to 65.37%, respectively, with corresponding average values of 33.47°C and 56.01%. The ambient temperature affects the ambient relative humidity; for example, a high ambient temperature results in a low ambient relative humidity and vice versa.

**Figure 7:**
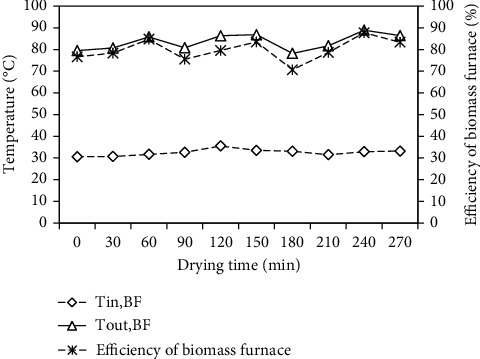
Variation of efficiency of biomass furnace and inlet and outlet temperatures of the biomass furnace with drying time.

The efficiency of the biomass furnace as well as the temperatures of air entering and leaving the biomass furnace with time is presented in Figure 7. These temperatures varied over 30.60–35.50°C and 78.20–88.90°C, with average values of 32.73 and 83.51°C, respectively. The efficiency of biomass furnace is the ratio of the extraction of heat energy from biomass furnace or heat energy used for drying process to the heat energy produced from burning of biomass fuel, and it was calculated using Eq. (18). The efficiency of the biomass furnace was found to be 70.63–87.70%, with an average value of 79.53% and an air mass flow rate of 0.1084 kgs^−1^.

**Figure 8:**
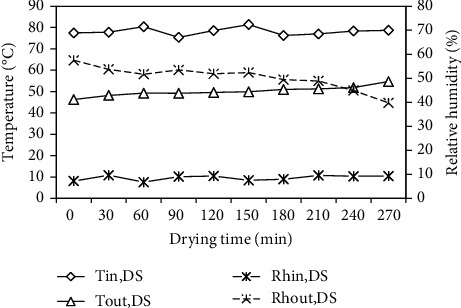
Variation of temperatures and relative humidity at the inlet and outlet of the drying section with drying time.

The air temperature and relative humidity values of air entering and leaving the drying section with the drying time are presented in Figure 8. The temperatures of air entering and leaving the drying section were in the ranges of 75.40–81.40°C and 46.30–54.70°C, respectively, with corresponding average values of 78.15°C and 50.14°C. The relative humidity values of air entering and leaving the drying section were in the ranges of 6.77%–9.57% and 39.75%–57.45%, with corresponding average values of 8.55% and 50.35%. As shown in Figure 8, the air temperature value leaving the drying section increased with increasing in drying time, while the air relative humidity value decreased with increasing in drying time.

These, due to the heat and mass transfer coefficients, decreased with increasing in the drying time.

**Figure 9:**
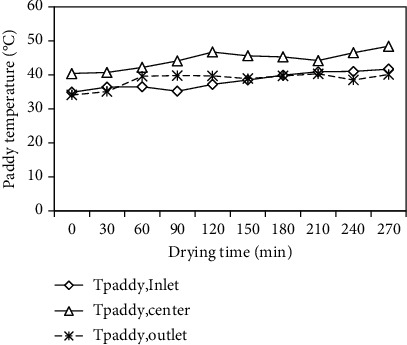
Variation of paddy temperatures at the inlet, center, and outlet of the drying section with drying time.

The paddy temperatures at the inlet, center, and outlet of the drying section with the drying time are illustrated in Figure 9. These temperatures varied from 34.90 to 41.70°C, 40.40 to 48.40°C, and 34.10 to 40.30°C, respectively, with corresponding average values of 38.22, 44.41, and 38.58°C. As shown in Figure 9, the paddy temperature at the center of the drying section was observed to be higher than the paddy temperatures at the inlet and outlet of the drying section due to high heat transfer rate in the drying section.

**Figure 10:**
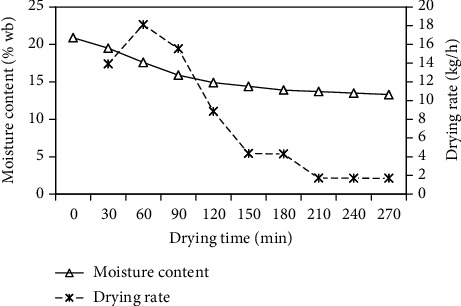
Variation of moisture content and drying rate with drying time.

The variation in the paddy moisture content and drying rate with the drying time is presented in Figure 10. The paddy moisture content inside the drying section was reduced from 20.90% wet basis (initial weight 400 kg) to 13.30% wet basis (final weight 364 kg) in 270 min, for a mass flow rate of 0.1084 kg/s, average temperature of 78.15°C, and average relative humidity of 8.55%. The drying rate is defined as the mass of moisture removed from the product per unit time and it was estimated using Eq. (6). The drying rate was found to be 1.688–18.126 kg/h, with an average of 7.792 kg/h. As shown in Figure 10, paddy drying occurred in the falling rate period, and constant drying rate period was not observed. In the falling rate period, the material surface is no longer saturated with water, and the drying rate is controlled by diffusion of moisture from the interior of solid to the surface. During the falling rate period, the drying rate decreased with increasing in drying time, and this is due to the decreased mass transfer rate with increasing in drying time.

**Figure 11:**
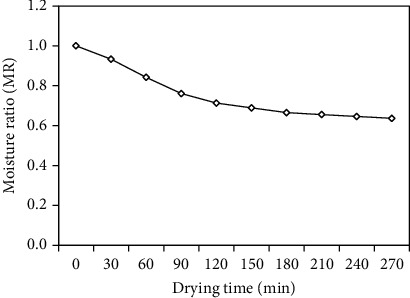
Variation of moisture ratio (MR) with drying time.

The variation of the moisture ratio (MR) with the drying time is illustrated in Figure 11. It is defined as the ratio of the initial moisture content of paddy to the instantaneous moisture content of paddy during the drying process, and it was calculated using Eq. (9). The moisture ratio of paddy in PSBA-RMFD decreased exponentially with increasing drying time. The continuous decrease in the moisture ratio indicates that diffusion has governed the internal mass transfer.

**Figure 12:**
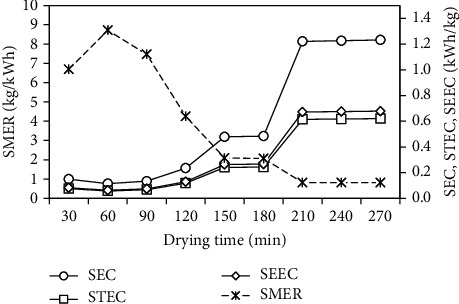
Variation of SMER, SEC, STEC, and SEEC with drying time.

The variation of the SMER with the drying time is presented in Figure 12. It is defined as the ratio of the moisture evaporated from wet product to the energy input to the drying system and it was calculated using Eq. (5). The SMER was in the range of 0.122–1.308 kg/kW h, with an average of 0.562 kg/kW h. Figure 12 also shows the variation of the SEC, STEC, and SEEC with the drying time. The SEC is the ratio of the total energy input (thermal energy + electrical energy) to the drying system to the moisture evaporated from wet material, the STEC is the ratio of the thermal energy input to the drying system to the moisture evaporated from wet material, the SEEC is the ratio of the electrical energy input to the drying system to the moisture evaporated from wet material, and these were calculated using Eqs. (2)–(4), respectively. The SEC ranged between 0.806 and 8.657 kW h/kg, with an average of 4.119 kW h/kg, while the STEC and SEEC ranged between 0.385 and 4.136 kW h/kg and 0.421 and 4.521 kW h/kg, respectively, with corresponding average values of 1.968 and 2.151 kW h/kg. As seen from Figure 12, the SMER decreased with increasing in drying time, while the SEC, STEC, and SEEC increased with increasing in drying time, and these is due to the decreased the moisture removal rate with increasing in drying time.

**Figure 13:**
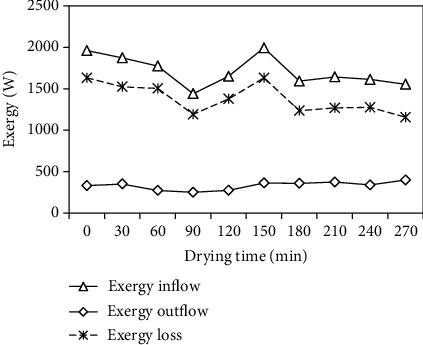
Variation of exergies with drying time.

The inflow, outflow, and loss of exergy for the drying section during the drying experiment versus the drying time are presented in Figure 13, and these were calculated using Eqs. (13)–(15), respectively. These three parameters were found to be over 1439.42–1959.34 W, 249.24–398.17 W, and 1154.94–1630.24 W, respectively, with corresponding average values of 1708.32, 330.08, and 1378.24 W.

**Figure 14:**
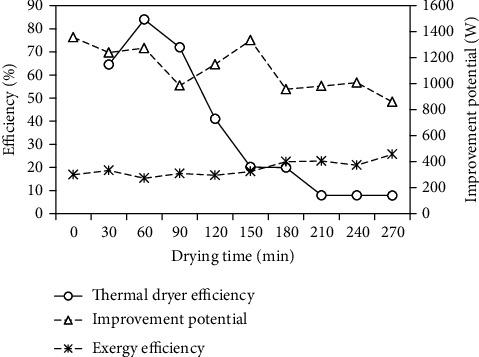
Variation of efficiencies and improvement potential with drying time.

The thermal dryer and exergy efficiencies of the PSBA-RMFD versus the drying time are presented in Figure 14. The thermal efficiency of the drying system is the ratio of the energy used for moisture evaporation to the energy input to the drying system, the exergy efficiency is the ratio of exergy use in the drying of the product to exergy of the drying air supplied to the drying chamber, and these were calculated using Eqs. (12) and (16), respectively. The thermal efficiency varied from 7.82% to 83.99%, with an average of 36.105%, while the exergy efficiency ranged from 15.28% to 25.64%, with an average of 19.46%. As shown in Figure 14, the thermal dryer and exergy efficiencies were found to be high at the beginning of the drying process and low at the end of the drying process. The high values of the thermal dryer and exergy efficiencies at the beginning of the drying process due to rapid evaporation of the surface moisture of the paddy or the drying rate is very high and vice versa. Furthermore, they decreases exponentially during the drying from inside the paddy until the end of the drying process. Figure 14 also shows the improvement potential of the dryer with the drying time and it was calculated using Eq. (17), which was in the range of 858.90–1355.62 W, with an average of 1114 W. A summary of the performance evaluation of the PSBA-RMFD is presented in Table 7.

### 3.2. Evaluation of the Effect of Drying Air Temperature on the Quality of Drying Products

In the drying process, beside performance of drying system and drying time, the quality of drying product must be taken into consideration, and the quality of drying product is greatly influenced by drying air temperature. The drying air temperature may cause the quality degradation of the drying product. In the paddy drying process, the quality of the drying product can be expressed in terms of color of paddy grains and percentages of head rice yield, broken rice, rice grouts, and rice of whitewash. The percentages of head rice, broken rice, rice grouts and rice of whitewash obtained by milling the paddy. In PSBA-RMFD, the paddy grains is dried with a drying air temperature of 75.40–81.40°C, and the moisture content of the paddy grains in the dryer is reduced from 20.90% wet basis to 13.30% wet basis in 270 minutes. The values of head rice yield, broken rice, rice grouts and rice of whitewash were 67.37 ±0.69%, 26.34 ±0.80%, 3.55 ±0.51%, and 2.72 ±0.75%, respectively. Based on the Indonesian national rice quality standard (SNI No.01-6128-2015) as shown in Table 8, the quality of rice obtained by drying paddy grains using the PSBA-RMFD at a drying air temperature of 75.40–81.40°C is belong to in the third medium class. It can be stated that the quality of rice is low or there is a decrease in the quality of paddy during drying process due to the low percentage of head rice yield and high percentages of broken rice, rice groats, and rice of whitewash.

**Figure 15:**
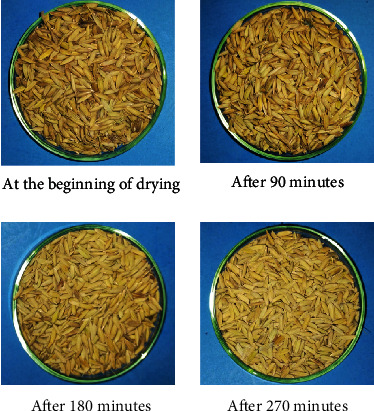
Color of the paddy grain samples after successive drying time intervals.

The color of the paddy grain samples after successive drying time intervals is shown in Figure 15. From the figure, it can be seen that the color of the paddy grain sample during the drying process at a drying air temperature of 75.40-81.40°C changed significantly. This is due to the high drying air temperature and longer drying time to achieve a paddy moisture content of around 13.3%.

### 3.3. Economic Evaluation

An economic analysis was performed for the PSBA-RFMD to calculate the PP and NPV, which are strongly determined by *I*_*C*_, *C*_pr_, total sale of the dried product, and PR. *I*_*C*_ of the PSBA-RMFD included the costs of buying the construction material and equipment and the labor cost for the construction and installation. The components of *I*_*C*_ of the PSBA-RFMD are listed in Table 4. The annual *C*_pr_ (drying cost) was classified into direct and fixed costs. The direct costs included the cost of buying the fresh paddy, labor cost, electricity cost, and biomass fuel cost; these costs were found to be 110288.87, 5036.02, 2337.77, and 1208.64 USD/year, respectively. The fixed costs included the maintenance cost and depreciation, and these were found to be 76.94 and 346.23 USD/year, respectively. Then, the annual *C*_pr_ (drying cost) was found to be 119294.47 USD/year. On the contrary, the annual PR and the *I*_*C*_ were 2003.34 and 3846.95 USD, respectively. Therefore, PP and NPV were found to be approximately 1.9 years and 7472.34 USD, respectively. A summary of the economic evaluation of the PSBA-RMFD is presented in Table 9.

## 4. Conclusions

A PSBA-RMFD with a drying capacity of 400 kg/h was tested and evaluated for its performance. The results showed that the PSBA-RMFD reduced the paddy moisture content from 20.90% (wet basis) to 13.30% (wet basis) in 270 min, with an average temperature of 78.15°C and average relative humidity of 8.55%. The SEC varied in the range of 0.806–8.657 kW h/kg of water evaporated, with an average of 4.119 kW h/kg of water evaporated. The values of the STEC and SEEC varied from 0.385 to 4.136 kW h/kg and 0.421 to 4.521 kW h/kg of water evaporated, respectively, with respective averages of 1.968 and 2.151 kW h/kg of water evaporated. The SMER was in the range of 0.122–1.308 kg of water evaporated/kW h, with an average of 0.562 kg/kW h. The values of the thermal dryer and exergy efficiencies were in the range of 7.82%–83.99% and 15.28%–25.64%, respectively, with corresponding averages of 36.11% and 19.46%.The IP was in the range of 858.90–1355.62 W, with an average of 1114 W. The efficiency of the biomass furnace varied between 70.63% and 87.70%, with an average of 79.53%. The PP achieved by the PSBA-RMFD was 1.9 years. The percentage of biomass energy used in this drying system was approximately 47.77% of the overall energy. In addition, the heat energy required for heating the drying air during the drying process was supplied only by the biomass fuel. However, the quality of paddy grains was changes significantly during drying process.

## Figures and Tables

**Table 1:** Summary of energy consumption of various types of dryers for paddy drying (from the literature).

No.	Type of dryer	Temperature (°C)	Moisture content (%) wb		SEEC (MJ/kg of water evaporated)	STEC (MJ/kg of water evaporated)	SEC (MJ/kg of water evaporated)	Reference
			Initial	Final				
1	Pilot-scale fluidized bed dryer, feed rate 1.0 t/h	100–120	30–32	19–19.7	—	—	1.587–2.418	[35]
2	Fluidized bed dryer, capacity (feed rate) 0.82 ton/h	100–120	45 (db)	24 (db)	0.53	1.79	—	[36]
3	Industrial-scale fluidized bed dryer, feed rate 2.5–4.0 t/h	144	25	20	—	—	4.2	[37]
4	Laboratory scale spouted bed dryer, feed rate 1000 kg/h	144	20 (db)	14.4 (db)	0.76	6.7	—	[44]
5	Industrial-scale fluidized bed dryer, feed rate 2.3–7.9 t/h	150	18–21.1	15.3–15.5	—	—	5.36–6.89	[38]
6	Fluidized bed dryer	100–110	21–23.6	10.4–13.6	—	—	3.874–4.421	[39]
7	Combined impinging stream and pneumatic drying system, mass flow rate (feed rate) 22 kg_dry solid_/h	70	28	21.4	2.2	0.9	3.1	[51]
8	Impinging stream dryer, particle flow rate (feed rate) 150 kg_dry solid_/h	110	28 (db)	21.7 (db)	—	—	5.1	[50]
9	Fluidized bed dryer	80	20	14	—	—	30.9	[40]
	Spouted bed dryer	80	20	14	—	—	11.7	
10	Industrial inclined bed dryer, holding capacity 15 t	41–42	22–23	12.5	1.44–195	2.77–3.47	—	[34]
11	Industrial fluidized bed dryer, capacity (feed rate) 7.75 t/h	100–120	36.98 (db)	27.58 (db)	0.79	7.57	—	[41]
12	Fully automated pilot-scale model fluidized bed dryer, feed rate 500 kg/h	70	28	14.1	—	—	2.84	[42]
13	Horizontal rotary dryer, holding capacity 3 t	38–40	14.5	8	5.50–17.41	11.5–36.44	—	[49]
14	Fluidized bed dryer (vertical), holding capacity 12 kg	78	20	14	—	—	2.27–7.69 kW h/kg	[17]
15	Fixed deep bed dryer	80	20.4	12	—	—	1.891–12.014 kW h/kg	[33]
16	Small scale mixed-flow dryer,holding capacity 30 kg	45	25	12	0.97	3.17	—	[47]
17	Industrial recirculating grain dryer, holding capacity 30 t	60	30	14	19.2 kW h/t	371.9 MJ/t	—	[48]
18	Fluidized bed dryer (horizontal), holding capacity 11 kg	59.58	20	14	1.960 kW h/kg	1.532 kW h/kg	3.528 kW h/kg	[18]
19	Crossflow dryer, feed rate 60 kg/h	130	20.6	14	—	—	5.85	[45]
20	Continuous crossflow dryer, feed rate 36 kg/h	150	20	14.3	—	—	3.6	[46]
21	Solar-assisted fluidized bed dryer (vertical), holding capacity 0.5 kg	45	21	10	—	—	8.30–22.12 kW h/kg	[43]
22	Rotary dryer, holding capacity 3000 kg	35–40	12	8	—	—	10.48	[32]
	Fixed bed dryer, holding capacity 3000 kg	30–40	12	7.6	—	—	9.81	

SEEC: specific electrical energy consumption; STEC: specific thermal energy consumption; SEC: specific energy consumption; db: dry basis; Wb: wet basis.

**Table 2:** Equations used to determine the performance of the PSBA-RMFD.

Performance indicator	Equation	Eq. no.	Ref.
Specific energy consumption	$\text{SEC}=E_{\text{bmf}}+E_{\text{Bbf}}+E_{\text{Mdr}}+E_{\text{Mbe}}+E_{\text{Mvf}}+E_{\text{Bmfd}}/{\dot{m}}_{\text{water}}$	(2)	[43]
Specific thermal energy consumption	$\text{STEC}=E_{\text{bmf}}/{\dot{m}}_{\text{water}}$	(3)	[18, 34]
Specific electrical energy consumption	$\text{SEEC}=E_{\text{Bbf}}+E_{\text{Mdr}}+E_{\text{Mbe}}+E_{\text{Mvf}}+E_{\text{Bmfd}}/{\dot{m}}_{\text{water}}$	(4)	[18, 34]
Specific moisture evaporation rate	$\text{SMER}={\dot{m}}_{\text{water}}/E_{\text{bmf}}+E_{\text{Bbf}}+E_{\text{Mdr}}+E_{\text{Mbe}}+E_{\text{Mvf}}+E_{\text{Bmfd}}$	(5)	[55]
Drying rate	${\dot{m}}_{\text{water}}=M_{\text{Cdb},t+\Delta \text{t}}-M_{\text{Cdb},t}/\Delta t$	(6)	[54]
Paddy moisture content (wet basis)	*M*_Cwb_ = *m*_wetpd_ − *m*_bonedrypd_/*m*_wetpd_	(7)	[54]
Paddy moisture content (dry basis)	*M*_Cdb_ = *m*_wetpd_ − *m*_bonedrypd_/*m*_bonedrypd_	(8)	[54]
Moisture ratio	MR = Mt/Mo	(9)	[43]
Electrical energies required by blower of biomass furnace, motor of bucket elevator, and motor of vibratory feeder	*E*_Bbf_, *E*_Mbe_, and*E*_Mvf_ = VIcos*ϕ*	(10)	[22]
Electrical energies required by the motor of discharge roller and blower of mixed-flow dryer	$E_{\text{Mdr}}\text{ and }E_{\text{Bmfd}}=\sqrt{3}\text{VIcos}\phi$	(11)	[17]
Dryer thermal efficiency	$\eta_{\text{th}}={\dot{m}}_{\text{water}}H_{\text{fg}}/E_{\text{bmf}}+E_{\text{Bbf}}+E_{\text{Mbe}}+E_{\text{Mvf}}+E_{\text{Bmfd}}$	(12)	[47]
Exergy inflow	${\text{EX}}_{i,\text{ds}}={\dot{m}}_{a}C_{\text{Pa}}\left[\left(T_{\text{ai},\text{ds}}-T_{\text{amb}}\right)-T_{\text{amb}}\ln\left(T_{\text{ai},\text{ds}}/T_{\text{amb}}\right)\right]$	(13)	[17, 53]
Exergy outflow	${\text{EX}}_{o,\text{ds}}={\dot{m}}_{a}C_{\text{Pa}}\left[\left(T_{\text{ao},\text{ds}}-T_{\text{amb}}\right)-T_{\text{amb}}\ln\left(T_{\text{ao},\text{ds}}/T_{\text{amb}}\right)\right]$	(14)	[17, 53]
Exergy losses	EX_loss_ = EX_*i*,ds_ − EX_*o*,ds_	(15)	[17, 53]
Exergy efficiency of drying section	*η*_Ex_ = EX_*o*,ds_/EX_*i*,ds_ = 1 − EX_loss_/EX_*i*,ds_	(16)	[17, 53]
Improvement potential	IP = (1‐*η*_Ex_)EX_loss_	(17)	[54]
Efficiency of biomass furnace	*η*_BF_ = (*E*_Ubf_/*E*_bmf_)*x*100%	(18)	[22]
Heat energy produced from burning of biomass fuel	$E_{\text{bmf}}={\dot{m}}_{\text{bmf}}{\text{CV}}_{\text{bmf}}$	(19)	[22]
Useful heat from biomass furnace	$E_{\text{Ubf}}={\dot{m}}_{\text{a}}C_{\text{Pa}}\left(T_{\text{ao},\text{bf}}-T_{\text{ai},\text{bf}}\right)$	(20)	[22]

**Table 3:** Data from the drying experiment, used for the performance analysis of the PSBA-RMFD.

Time (min)	Moisture content (%,wet basis)	Temperature (°C)											Relative humidity (%)		
		T1	T2	T3	T4	T5	T6	T7	T8	T9	T10	T11	Rh_amb_	Rh_*i*,ds_	Rh_*o*,ds_
0	20.90	30.30	25.00	30.60	79.50	77.50	35.50	46.30	37.50	34.90	40.40	34.10	65.37	7.22	57.45
30	19.50	31.40	25.50	30.70	80.70	77.80	38.20	48.20	38.20	36.40	40.70	35.10	62.63	9.64	53.72
60	17.60	34.20	26.10	31.70	85.80	80.40	36.40	49.30	38.60	36.50	42.20	39.60	53.04	6.77	51.69
90	15.90	34.70	27.20	32.60	80.80	75.40	36.30	49.20	39.00	35.20	44.10	39.80	56.39	9.02	53.49
120	14.90	34.40	25.80	35.50	86.30	78.60	38.30	49.60	38.90	37.20	46.70	39.70	50.76	9.31	51.85
150	14.40	32.50	25.40	33.50	86.80	81.40	37.80	49.90	39.30	38.50	45.60	38.90	56.86	7.53	52.38
180	13.90	33.50	25.90	33.10	78.20	76.30	35.70	51.00	39.40	39.90	45.30	39.70	55.01	7.97	49.41
210	13.70	33.40	25.70	31.50	81.70	77.00	37.70	51.30	39.50	40.90	44.20	40.30	54.43	9.57	48.88
240	13.50	34.70	26.40	32.90	88.90	78.40	38.10	51.90	38.80	41.00	46.50	38.50	52.45	9.22	44.89
270	13.30	35.60	27.30	33.20	86.40	78.70	38.30	54.70	39.40	41.70	48.40	40.10	53.13	9.26	39.75

Rh_amb_: ambient relative humidity; Rh_i,ds_: relative humidity entering the drying section; Rh_o,ds_: relative humidity leaving the drying section.

**Table 4:** Investment cost of the PSBA-RMFD.

Item	USD
Biomass furnace	314.75
Drying column	649.09
Bucket elevator	669.02
Vibratory feeder	220.67
Air duct	174.86
Blower	909.28
Labor for construction and installation	909.28
Total	3,846.95

**Table 5:** Equations used to determine the production cost, profit, payback period, and net present value of the PSBA-RMFD.

Economic parameter	Equation	Eq. no.	Ref.
Production cost	*C*_Pr_ = *C*_fm_ + *C*_ec_ + *C*_lb_ + *C*_ad_	(21)	[54, 56]
Energy consumption cost	*C*_ec_ = *C*_el_ + *C*_bmf_	(22)	[54]
Additional cost	*C*_ad_ = *C*_*m*_ + *C*_dp_	(23)	[54]
Profit	PR = TS − *I*_*C*_ − *C*_Pr_	(24)	[54]
Return of capital	ROC = PR/*I*_*C*_	(25)	[54]
Payback period	PP = *I*_*C*_/PR	(26)	[57]
Net present value	NPV = ∑_*n*−1_^*N*^*P*_*n*_(1 + *i*)^*n*^ − *I*_*C*_	(27)	[57]
Discounted present value (*S*)	*P*_*n*_ = *S*(1 + *i*)^−*n*^	(28)	[57]

**Table 6:** Uncertainties of parameters during the paddy drying experiment.

Parameter	Unit	Uncertainty range
Time measurement	Min	±0.1
Air temperature	°C	±0.20
Paddy temperature	°C	±0.20
Relative humidity	%	±0.346
Paddy moisture content change	% (wet basis)	±0.51
Mass of paddy	kg	±0.14
Mass of coconut shell charcoal	kg	±0.11
Air velocity	m/s	±0.24

**Table 7:** Performance evaluation of the PSBA-RMFD.

Parameter	Unit	Value
Initial moisture content (wet basis)	%	20.90
Final moisture content (wet basis)	%	13.30
Average drying air temperature	°C	78.15
Average drying air relative humidity	%	8.55
Drying time	Min	270
Average SMER	kg/kW h	0.56
Average SEC	kW h/kg	4.119
Average STEC	kW h/kg	1.968
Average SEEC	kW h/kg	2.151
Average thermal efficiency of the PSBA-RMFD	%	36.17
Average exergy efficiency	%	19.46
Average improvement potential	W	1114
Average efficiency of biomass furnace	%	79.53
Percentage of biomass energy used in the PSBA-RMFD	%	47.77

**Table 8:** Indonesian national rice quality standard (SNI No.01-6128-2015) [58].

No.	Quality components	Quality class			
		Premium	Medium		
			1	2	3
1	Level of polishing (%, min)	100	95	90	80
2	Moisture content (%, max)	14	14	14	15
3	Head rice (%, min)	95	78	73	60
4	Broken (%, max)	5	20	25	35
5	Grain grouts (%, max)	0	2	2	5
6	Red grain (%, max)	0	2	3	3
7	Yellow/damaged grain (%, max)	0	2	3	5
8	Grain of whitewash (%, max)	0	2	3	5
9	Foreign object (%, max)	0	0.02	0.05	0.2
10	Grains (grains/100 g, max	0	1	2	3

**Table 9:** Economic evaluation of PSBA-RMFD.

Parameter	Unit	Value
Investment cost (initial cost) (*I*_*C*_)	USD	3,846.95
Initial weight of paddy	kg	400
Final weight of paddy	kg	364
Price of fresh paddy	USD/kg	0.385
Price of dried paddy	USD/kg	0.462
Price of coconut shell charcoal	USD/kg	0.105
Price of electricity	USD/kW h	0.095
Labor charge	USD/day	6.99
Discount factor	%	12
Cost of maintenance	%	2
Depreciation	%	10
Life of the PSBA-RMFD	Year	10
Annual production cost (*C*_pr_)	USD/year	119,294.47
Annual total sale of dried product (revenue)	USD/year	121,297.82
Annual profit	USD/year	2,003.34
Payback period (PP)	Year	1.9
Net present value (NPV)	USD	7,472.34

## Data Availability

The data from drying experiment can be access at the following link: https://drive.google.com/file/d/1pgSOLhNxs63UI785y-gMhXwg6Oe_a_vF/view?usp=sharing. And also the data from drying experiment is already in the manuscript and can be seen in Table 3.

## Nomenclature

### Abbreviations

*C*_ad_: Additional cost
*C*_bmf_: Biomass fuel cost
*C*_dp_: Depreciation cost
*C*_ec_: Energy consumption cost
*C*_el_: Electricity cost
*C*_fm_: Fresh material cost
*C*_lb_: Labor cost
*C*_*m*_: Maintenance cost
*C*_Pa_: Air specific heat (Jkg^−1^C^−1^)
*C*_Pr_: Production cost
CV_bmf_: Biomass fuel caloric value (kcal/kg)
cos*ϕ*: Power factor
*E*_Bbf_: Electrical energy required by the biomass furnace blower (W)
*E*_bmf_: Heat energy produced from burning of the biomass fuel (W)
*E*_Bmfd_: Electrical energy required by the mixed flow dryer blower (W)
*E*_Mbe_: Electrical energy required by the bucket elevator motor (W)
*E*_Mdr_: Electrical energy required by the discharge roller motor (W)
*E*_Mvf_: Electrical energy required by the vibratory feeder motor (W)
*H*_fg_: Latent heat of vaporization of water (kJ/kg)
*I*: Current (A)
${\dot{m}}_{a}$: Mass flow rate of air (kg/s)
${\dot{m}}_{\text{bmf}}$: Consumption rate of biomass fuel (kg/h)
*m*_bonedrypd_: Mass of bone dry of paddy (kg)
*M*_Cdb,*t*_: Paddy moisture content (dry basis) at the time “*t*”
*M*_Cdb,*t*+Δt_: Paddy moisture content (dry basis) at the time “t + Δt”
*M*_*o*_: Initial paddy moisture content (%)
*M*_*t*_: Instantaneous paddy moisture content (%)
${\dot{m}}_{\text{water}}$: Drying rate (kg/h)
*m*_wetpd_: Mass of wet of paddy (kg)
Rh_amb_: Ambient relative humidity (%)
Rh_*i*,ds and_ Rh_*o*,ds_: Relative humidity entering and leaving the drying section, respectively (%)
T1_=_*T*_amb (db)_: Dry bulk ambient temperature (°C)
T2_=_*T*_amb (wb)_: Wet bulk ambient temperature (°C)
T3_=_*T*_ai,bf_: Air temperature at the inlet of the biomass furnace (dry bulk) (°C)
T4_=_*T*_ao,bf_: Air temperature at the outlet of the biomass furnace (dry bulk) (°C)
T5_=_*T*_ai,ds (db)_: Temperature of dry bulk air entering the drying section (°C)
T6_=_*T*_ai,ds (wb)_: Temperature of wet bulk air entering the drying section (°C)
T7_=_*T*_pdi,ds_: Paddy temperature at the inlet of the drying section (°C)
T8_=_*T*_pdc,ds_: Paddy temperature at the center of the drying section (°C)
T9_=_*T*_pdo,ds_: Paddy temperature at the outlet of the drying section (°C)
*T*_10=_ *T*_ao,ds (db)_: Dry bulk air temperature at the outlet of the drying section (°C)
*T*_11=_ *T*_ao,ds (wb)_: Wet bulk air temperature at the outlet of the drying section (°C)
*V*: Voltage (V)
Δ*t*: Interval of drying time (h).

## Additional Points

*Highlights.* (i) A pilot-scale biomass-assisted recirculating mixed-flow drying system for drying paddy (PSBA-RMFD) was tested. (ii) The average specific energy consumption (SEC), specific thermal energy consumption (STEC), and specific thermal energy consumption (SEEC) were 4.119, 1.968, and 2.151 kW h/kg of water evaporated, respectively. (iii) The average thermal dryer efficiency was 36.11%. (iv) The average exergy efficiency and payback period of the dryer were 19.46% and 1.9 years, respectively. (v) The heat energy required in the drying process was supplied only by biomass energy
